# The impact of COVID-19 pandemic on Islamic versus conventional stock markets: international evidence from financial markets

**DOI:** 10.1186/s43093-021-00078-5

**Published:** 2021-09-08

**Authors:** Naji Mansour Nomran, Razali Haron

**Affiliations:** 1 Present Address: Department of Banking and Finance, Faculty of Administrative and Financial Sciences, University of Saba Region, Marib, Yemen; 2 Department of Finance and Banking, Faculty of Administrative Sciences, Thamar University, Thamar, Yemen; 3 IIUM Institute of Islamic Banking and Finance (IIiBF), International Islamic University Malaysia, Kuala Lumpur, Malaysia; 4 Present Address: International Islamic University Malaysia, Jalan Gombak, 53100 Kuala Lumpur, Malaysia

**Keywords:** Financial markets, Islamic stock market, Conventional stock market, COVID-19, Coronavirus, Pandemic

## Abstract

This study employs sample *t*-tests and panel pooled OLS regression to investigate the impact of COVID-19 pandemic on Islamic versus conventional stock markets returns. The study uses daily data from 15 countries over the period of September 01, 2019–April 30, 2020, which covers two main periods and over four sub-periods. Findings reveal that the returns of Islamic indices begun to be positive instead of negative by mid-April 2020, while returns of conventional ones remain negative throughout the periods. Furthermore, the results suggest a negative and statistically significant impact of COVID-19 on the performance of both stock indices. Nevertheless, this impact is weak on the Islamic indices and strong on the conventional ones. Overall, the findings indicate that Islamic stock markets perform better before and during COVID-19 than the conventional ones, and the adverse impact of the pandemic on the stock markets is relatively lesser for the Islamic indices.

## Introduction

The current century has witnessed different global epidemics, e.g. the SARS, virus H7N9, Ebola virus, and COVID-19 pandemic. Most of the studies that have examined the impact of these epidemics on the performance of stock markets worldwide found a negative impact [2]. Regarding the current pandemic, i.e. COVID-19, Goodell [10] states that this pandemic has resulted in destructive impacts on the global economic as a whole. The pandemic, which has spread to 216 countries globally, affects different aspects of the global economy including the stock markets [6, 21]. More specifically, the performance of stock markets globally was affected by the pandemic [19]. Inevitably, the pandemic caused an excessive level of risk, which in turn reflected in significant loses for investors over very short period [23]. Aggravating to the problem, the second wave of pandemic appeared in some countries, indicating that there is no solution to the pandemic yet [14]. Thus, a high degree of uncertainty would remain as the pandemic continues [23].

Parallel to the remarkable spread of COVID-19 pandemic, many studies have been conducted to evaluate its impact on the economy in general and on the stock markets in particular. In this regard, Sherif [19] reviews many studies and highlights that decisions related to the performance of Islamic and conventional stock market indices are very important empirical question, especially during the pandemic. As Sherif [19] indicates, investors prefer to invest with escalated profits, and then, the Islamic stock indices are more efficient and competitive compared to the conventional ones. During crises facing financial systems like the current pandemic, information about Islamic investment structures has become a subject of interest by investors who consider stock market returns as being uncertain.

As explained in the study of Saiti et al. [16], it is believed that Islamic stock indices are more resilient to a financial crisis compared to their conventional counterparts. It is important to mention that *Shari’ah* principles prevent trading cash as an asset. Basically, Islamic indices only include the financial sectors associated with supportive activities, hence making the Islamic products less risky as it is backed by real economic activities. According to Sherif [19], there are indeed differences between Islamic and conventional indices in terms of screening and financial characteristics other than the *Shari’ah* compliance investments. The low leverage and low account receivables are the financial characteristics of component stocks of Islamic indices, which in turns decrease the financial risks and the vulnerabilities, associated with crisis periods such as COVID-19. For that, this study aims to empirically investigate and compare the impact of COVID-19 on the Islamic stock markets versus conventional ones globally, responding to the lack of studies on this issue as mentioned by Sherif [19].

The remainder of this study is as follows: “Review of the literature” section discusses the related literature. “Data and methodology” section presents the data and methodology. “Results and discussion” section presents the empirical results and discussion while “Conclusion” section provides the concluding remarks.

## Review of the literature

Generally, several studies examined the impact of COVID-19 pandemic on stock markets performance and conclude that daily growth in confirmed cases due to COVID-19 negatively affected stock returns [1, 2, 4, 5, 21, 23]. By reviewing the current studies that link the pandemic to the stock markets, it is noticed that most of them focus on the stock indices in general and neglect the difference between the Islamic and conventional indices. This is essential because by distinguishing between the two, the behaviour and performance of the indices could be ascertained, especially during the pandemic crisis, which is unprecedented. This may assist related parties, e.g. policy makers and market investors for policy implementation and investment strategies. The current study therefore aims to fill the research gap in this aspect.

To the best of our knowledge, however, it seems that the exception is the studies of Sherif [19], Erdoğan et al. [9], Salisu and Sikiru [17], Yarovaya et al. [22] and Arif et al. [3]. Nevertheless, all of these studies suffer from some limitations. Sherif [19] examines the impact of pandemic on the Islamic UK Dow Jones index versus its UK counterpart while Erdoğan et al. [9] examine the impact of pandemic on the Islamic and conventional stock markets in Turkey. Both studies of Sherif [19] and Erdoğan et al. [9] suffer from the same limitations. First, the studies are country specific and only examine the issue in the UK and Turkey stock markets; thereby, their findings cannot be generalized to the other markets across countries. Second, they examine the impact of pandemic on the mentioned UK and Turkey indices over one period as many studies do, which seems to be not enough to evaluate the impact of COVID-19. In this regard, Topcu and Gulal [21] examine the impact of pandemic on emerging stock markets and they divide the sample period during COVID-19 into three sub-samples to understand how the impact of the pandemic changed over time.

Salisu and Sikiru [17] examine whether the two composite stock indices, DJIM and DJCA^1^ as proxies for the Asia–Pacific Islamic and the conventional stock price indices, respectively, can serve as good hedge against uncertainty due to pandemics and epidemics. On average, they find evidence of higher hedging potential for the Asia–Pacific Islamic stocks against uncertainty relative to the conventional one. Yarovaya et al. [22] also examine the impact of the pandemic on spillover between conventional and Islamic stock and bond markets indices of Dow Jones Market^2^, and they provide empirical evidence on safe haven properties of Islamic stocks and Islamic bonds (Sukuk), during the pandemic. They find that the spillovers between conventional and Islamic stock markets become stronger during the pandemic, while Sukuk can be used as a hedge of conventional bond markets during the pandemic. Recently, Arif et al. [3] explore Islamic stocks’ safe-haven properties against G7 conventional counterparts using the cross-quantilogram model and dataset consist of the Dow Jones Islamic world market (DJIM) index, MSCI G7 group, and individual country indices to proxy for Islamic and conventional equity investments, respectively. They find that Islamic stocks emerged as a robust safe-haven asset for the G7 stock markets during the pandemic.

It is clear from the studies of Salisu and Sikiru [17], Yarovaya et al. [22] and Arif et al. [3] that they almost share the similar objective which is examining whether Islamic stocks serve as safe-haven assets during the pandemic. The main differences between these studies may lie in the used dataset and methods employed. The dataset and method used by Salisu and Sikiru [17] consists of two composite stock indices and a predictability method, while those used by Yarovaya et al. [22] consist of Dow Jones Market indices and the VARMA-BEKK-AGARCH method, and lastly, those used by Arif et al. [3] consist of the Dow Jones Islamic world market (DJIM) index, MSCI G7 group, and individual country indices and the cross-quantilogram method. However, apparently none of these studies examine the impact of the pandemic on Islamic versus conventional stock markets returns using the techniques employed in the present study. Indeed, there is a lack of studies that examine how the Islamic stock market indices perform during the COVID-19, which is a significant issue that still requires more empirical studies [19]. Thus, the aim of this study is to empirically investigate and compare the impact of COVID-19 on the return of Islamic stock market indices versus conventional indices globally.

Based on the foregoing, this study offers the following contributions to the literature. First, this study is not country specific as conducted in most of the existing studies, e.g. [19]; it provides an international evidence by investigating the impact of COVID-19 pandemic on Islamic versus conventional stock markets returns across 15 countries and therefore draws a global conclusion. Second, the study conducts the analysis over different periods, overall, before, and during the COVID-19 pandemic. Moreover, it divides the period during the pandemic into four sub-periods and thus offers further analysis to evaluate the changing impact of the pandemic over time. Lastly, unlike most of the past studies, this study employs sample *t*-tests besides the panel pooled Ordinary Least Squares (OLS) regression technique to analyse the data and compare the performance of stock market indices during and before the pandemic. In addition, it is also to assess whether the means of return of each type of the indices (Islamic and conventional) during the pandemic are statistically different from its means before the pandemic.

## Data and methodology

### Sample construction and data collection

This study uses the daily data on Islamic and conventional stock market indices of 15 countries.^3^ The sample period is September 01, 2019, through April 30, 2020, which covers different time periods, before, and during COVID-19 pandemic.^4^ The study examines this whole sample period (Panel A: Overall period) as well as two sub-periods which are: Panel A_1_ (from September 01, 2019, to the day before 1st COVID-19 case was confirmed in a country) which represents the period before COVID-19 pandemic and Panel A_2_ (from the day when 1st COVID-19 case was confirmed in a country to April 30, 2020) which represents the period during COVID-19 pandemic. In order to provide pairwise comparison, two points were taken into consideration. First, both the Islamic and its conventional indexes counterpart were selected. However, except for Turkey and the UAE where the Islamic and its conventional counterpart are not available, the major active Islamic and conventional indices, which are the common market benchmarks used in most of the existing literature, are adopted instead. Second, the period of study was restricted to similar duration for both Islamic and conventional indices following prior studies, e.g. [12]. This resulted in getting similar number of daily observations for each pair of indices (Islamic vs. conventional) from each country (Panel A: 2416 vs. 2416; Panel A_1_: 1641 vs. 1641; Panel A_2_: 775 vs. 775) as shown in Table 1. The table reports the sample countries, the list of stock indices, and the date of 1st COVID-19 confirmed case across the countries for each panel.

**Table 1:** List of Islamic and conventional stock market indices from each country

No	Country	Index		The day when 1st COVID-19 case was confirmed*	Observations		
					Panel (A)	Panel (A_1_)	Panel (A_2_)
1	Bangladesh	Islamic	DSEX Shariah	Mar 09, 2020	143	130	13
		Conventional	DSEX		143	130	13
2	Canada	Islamic	S&P/TSX 60 Shariah	Jan 26, 2020	167	100	67
		Conventional	S&P/TSX 60		167	100	67
3	China	Islamic	FTSE Shariah China	Jan 22, 2020**	159	101	58
		Conventional	FTSE China		159	101	58
4	India	Islamic	FTSE Shariah India	Jan 30, 2020	173	107	66
		Conventional	FTSE India		173	107	66
5	Indonesia	Islamic	Jakarta Islamic Index	Mar 02, 2020	168	126	42
		Conventional	Jakarta SE Composite Index		168	126	42
6	Japan	Islamic	FTSE Shariah Japan 100	Jan 22, 2020	167	99	68
		Conventional	FTSE Japan		167	99	68
7	Kuwait	Islamic	FTSE Lujain Kuwait Shariah	Feb 24, 2020	136	98	38
		Conventional	FTSE Lujain Kuwait		136	98	38
8	Malaysia	Islamic	FTSE Bursa Malaysia Hijrah-Shariah	Jan 25, 2020	167	99	68
		Conventional	FTSE KLCI		167	99	68
9	Nigeria	Islamic	NSE Lotus Islamic	Feb 28, 2020	167	124	43
		Conventional	NSE 30		167	124	43
10	Pakistan	Islamic	KMI 30	Feb 27, 2020	169	124	45
		Conventional	KSE 100		169	124	45
11	Qatar	Islamic	QE Al Rayan Islamic	Mar 01, 2020	170	126	44
		Conventional	QE General		170	126	44
12	Taiwan	Islamic	FTSE TWSE Taiwan Shariah	Jan 25, 2020	159	96	63
		Conventional	FTSE Taiwan		159	96	63
13	Thailand	Islamic	FTSE SET Shariah	Jan 22, 2020	166	96	70
		Conventional	SET Index		166	96	70
14	Turkey	Islamic	KATILIM 50	Mar 12, 2020	171	136	35
		Conventional	BIST 100		171	136	35
15	UAE	Islamic	FTSE NASDAQ Dubai 10 Shariah	Jan 27, 2020	134	79	55
		Conventional	Dubai Financial Market General Index		134	79	55
Total		Islamic			2416	1641	775
		Conventional			2416	1641	775

Panel (A) represents the overall period (September 01, 2019–April 30, 2020); Panel (A_1_) represents the period before COVID-19 pandemic (September 01, 2019—the day before 1st COVID-19 case was confirmed in a country); Panel (A_2_) represents the period during COVID-19 pandemic (the day when 1st COVID-19 case was confirmed in a country—April 30, 2020); *This data is collected from the website of EU Open Data Portal; **The day the issue caught public eye, although China had cases well before Jan 22, 2020 (see, Ashraf [4, 5]; Similarly, Ashraf [4, 5] considered the same date Jan 22, 2020 as the 1st COVID-19 case was confirmed in Japan and Thailand; UAE denotes United Arab Emirates

The data are obtained from different sources: First, data on Islamic and conventional stock returns were collected from the website of www.investing.com.^5^ Second, data on daily COVID-19 confirmed cases^6^ for the countries were collected from the website of EU Open Data Portal [7]. Third, data of country-level control variables (democratic accountability, uncertainty avoidance, investment freedom, and GDP) were collected from different sources as given in Appendix 1 which also reports their definitions. After collecting the data, the daily COVID-19 data were appended with daily Islamic and conventional stock market returns data for Panel A_2_ which represents the COVID-19 pandemic period.

Furthermore, to understand how the impact of the pandemic changed over time and to provide robustness tests to confirm the results further, an additional analysis was conducted by dividing the sample period during COVID-19 pandemic, i.e. Panel A_2_, into four sub-periods, as provided in Table 6, which are: First sub-sample (March 12, 2020–March 31, 2020), Second sub-sample (March 12, 2020–April 10, 2020), Third sub-sample (March 12, 2020–April 17, 2020), and Fourth sub-sample (March 12, 2020–April 30, 2020). It is vital to illuminate the data for all the four sub-periods starting from March 12, 2020, as this date is the first day when all the 15 countries in the sample reported at least one positive case, besides the WHO officially declared COVID-19 as a global pandemic on March 11, 2020, as conducted by Topcu and Gulal [21].

### Methods

This section discusses the two main empirical methods used to examine and compare the impact of pandemic on the return of Islamic stock market indices and their conventional counterparts. These methods are paired sample *t*-tests and panel pooled OLS regression techniques.

Paired sample *t*-tests were applied to evaluate whether the means of return of the pairs of indices (Islamic vs. conventional indices) are statistically different from each other over the three main periods, overall, before, and during COVID-19 pandemic, besides the four sub-periods. Furthermore, sample *t*-tests were applied to assess whether the means of return of each type of the indices (Islamic and conventional) during the pandemic are statistically different from its means before the pandemic.

Following the existing literature, e.g. [4, 5, 21], the study also employs the panel pooled OLS regression with the heteroskedasticity robust standard errors to investigate the impact of growth in COVID-19 confirmed cases on both, Islamic and conventional stock market returns during the pandemic period. Thus, the return of Islamic and conventional stock market indices is given as a function of growth rate of COVID-19 confirmed cases, country-level control variables, weekly and country fixed-effects dummy variables as follows:

1 $$Y_{{C,t}} = \alpha _{C} + \beta _{{11}} {\text{COVID-19}}_{{C,t( - 1)}} + \mathop \sum \limits_{{K = 1}}^{K} \beta _{k} X_{{kC,t}} + \mathop \sum \limits_{{t = 1}}^{{T - 1}} \in _{t} W_{t} + \in _{{C,t}}$$

2 $$Y_{{C,t}} = \alpha _{C} + \beta _{{11}} {\text{COVID-19}}_{{C,t( - 1)}} + \mathop \sum \limits_{{t = 1}}^{{T - 1}} \in _{t} W_{t} + \mathop \sum \limits_{{t = 1}}^{{T - 1}} \in _{t} C_{t} + \in _{{C,t}}$$

where the *c* and *t* subscripts show country and day, respectively. *α*_*c*_ is a constant term. Dependent variable, *Y*, shows total Islamic(conventional) stock market returns in country *c* on day *t*. Islamic (conventional) stock market return is measured as the daily change in the stock market index of a country. COVID-19 shows the daily growth in COVID-19 confirmed cases. *X*_*kc,t*_ is a vector of country-level control variables which are democratic accountability, uncertainty avoidance, investment freedom, and log GDP. All of the mentioned country-level variables are employed to control for the variation in stock market returns across countries that differ in their institutional and macroeconomic conditions (see, [4]). *W*_*t*_ is a set of weekly fixed-effects dummies that control for weekly international factors (see, [8]). These dummies control for systematic risk. *C*_t_ is a set of country fixed-effects dummy variables. *Ɛ*_*c,t*_ is an error term. As mentioned above, this study uses heteroskedastic-robust standard errors to estimate *p*-values in regressions following [4].

## Results and discussion

The descriptive statistics of the main variables of the study for the overall period (Panel A) and for the two main sub-periods before and during the pandemic (Panel A_1_ and Panel A_2_) are presented in Table 2. For the overall period, Panel A of Table 2 shows that the mean values of the Islamic and conventional stock market returns are − 0.006 and − 0.042, respectively. This means, on average, the returns for both the indices are negative, but the Islamic indices are less negative compared to the conventional indices. As the minimum and maximum values show, Islamic stock indices returns swung between − 17.640 and + 12.810%, while the conventional indices swung between − 17.440 and + 12.390%. Likewise, for the period before the pandemic, Panel A_1_ of Table 2 presents that the mean values of the Islamic and conventional stock market returns are + 0.051 and + 0.042, respectively, which reflects that, on average, the returns for both the indices are positive, but the Islamic indices demonstrate higher positive than the conventional ones. Also, the minimum and maximum values show that Islamic stock market indices swung between − 5.360 and + 6.090%, while the conventional indices swung between − 5.540 and + 5.600%.

**Table 2:** Descriptive statistics of main variables

Variable	Observations	Mean	SD	Minimum value	Maximum value
*Panel (A): overall period*					
Islamic stock market returns (%)	2416	− 0.006	1.788	− 17.640	12.810
Conventional stock market returns (%)	2416	− 0.042	1.744	− 17.440	12.390
*Panel (A*_*1*_*): before COVID-19 pandemic*					
Islamic stock market returns (%)	1641	0.051	0.962	− 5.360	6.090
Conventional stock market returns (%)	1641	0.042	0.894	− 5.540	5.600
*Panel (A*_*2*_*): during COVID-19 pandemic*					
Islamic stock market returns (%)	775	− 0.129	2.828	− 17.640	12.810
Conventional stock market returns (%)	775	− 0.221	2.783	− 17.440	12.390
Growth in confirmed cases	564	1.554	6.884	0	156
Democratic accountability	775	3.933	1.422	1.500	6.000
Uncertainty avoidance	775	58.949	17.797	30.000	92.000
Investment freedom	775	54.329	14.993	20.000	80.000
Log (GDP)	775	12.332	0.516	11.515	13.469

Lastly, for the period during the pandemic, Panel A_2_ of Table 2 shows that the mean values of the Islamic and conventional stock market returns are − 0.129 and − 0.221, respectively, which reflects that, on average, the returns for both the indices are negative, but the Islamic indices are less negative compared to the conventional ones. Also, the minimum and maximum values show that Islamic stock market indices swung between − 17.640 and + 12.810%, while the conventional indices swung between − 17.440 and + 12.390%. In addition, Panel A_2_ reports that the mean of daily growth in pandemic confirmed cases is 155% with a standard deviation of 688%.

In short, Table 2 reports that Islamic indices outperformed the conventional ones over all the three different periods, overall, before, and during the COVID-19 pandemic, on average, for all the countries.

The analysis on paired t-test for the differences between the means of return of Islamic and conventional indices for each country and all the countries over the three main periods is shown in Table 3. For the overall period, Panel A of Table 3 indicates that there are significant differences in the average returns between Islamic and conventional indices for Taiwan and Turkey at 10% and 1% significance level, respectively, but not for the rest of indices from the other countries. According to Panel A, the mean values of the Islamic and conventional stock returns in Taiwan are − 0.010 and + 0.048, respectively, which reflects that, on average, the return of Islamic index is statistically less compared to the conventional one. In contrast, the mean values of the Islamic and conventional stock market returns in Turkey are + 0.234 and + 0.041, respectively, which reflects that, on average, the return of Islamic index is very strong and statistically higher than the conventional one. On average, Panel A reports that there is significant difference in the average returns between Islamic and conventional indices at 5% significance level for all the countries. As mentioned above, the mean values of the Islamic and conventional stock market returns for all the countries are − 0.006 and − 0.042, respectively, which means that, on average, their returns are negative, but the Islamic indices are strong and statistically less negative compared to the conventional ones.

**Table 3:** Summary statistics of Islamic and conventional stock market returns (%) across countries

Country	Index	Panel (A): overall period				Panel (A_1_): before COVID-19 pandemic				Panel (A_2_): during COVID-19 pandemic				*t*-test during versus before (periods)	
														*t*-value	*(p*-value)
		Obs	Mean	SD	Paired	Obs	Mean	SD	Paired	Obs	Mean	SD	Paired *t*-test	Is-during versus Is-before Co-during versus co-before	
					*t*-test				*t*-test				Is versus Co		
					Is versus Co				Is versus Co						
Bangladesh	Islamic	143	− 0.162	1.658	− 0.250	130	− 0.124	1.104	0.095	13	− 0.540	4.389	− 1.134	− 0.862	(0.389)
	Conventional	143	− 0.154	1.621	(0.802)	130	− 0.127	1.051	(0.924)	13	− 0.430	4.374	(0.278)	− 0.642	(0.521)
Canada	Islamic	167	0.033	2.099	0.810	100	0.061	0.610	− 0.117	67	− 0.007	3.243	0.886	− 0.205	(0.837)
	Conventional	167	− 0.027	2.451	(0.419)	100	0.065	0.375	(0.906)	67	− 0.166	3.856	(0.378)	− 0.599	(0.549)
China	Islamic	159	0.127	1.371	0.710	101	0.126	0.864	− 0.552	58	0.128	1.976	0.902	0.008	(0.993)
	Conventional	159	0.064	1.608	(0.478)	101	0.148	0.910	(0.582)	58	− 0.082	2.383	(0.370)	− 0.871	(0.385)
India	Islamic	173	0.010	2.292	1.046	107	0.129	1.078	0.884	66	− 0.183	3.456	0.631	− 0.873	(0.383)
	Conventional	173	− 0.036	2.066	(0.296)	107	0.090	0.857	(0.378)	66	− 0.242	3.167	(0.529)	− 1.028	(0.305)
Indonesia	Islamic	168	− 0.128	2.271	0.539	126	− 0.167	0.995	− 1.217	42	− 0.009	4.239	1.465	0.389	(0.697)
	Conventional	168	− 0.160	1.733	(0.590)	126	− 0.115	0.739	(0.225)	42	− 0.294	3.246	(0.150)	− 0.578	(0.564)
Japan	Islamic	167	0.004	1.453	0.668	99	0.166	0.679	1.878*	68	− 0.232	2.113	− 0.300	− 1.756*	(0.080)
	Conventional	167	− 0.006	1.435	(0.505)	99	0.141	0.666	(0.063)	68	− 0.222	2.093	(0.764)	− 1.614	(0.108)
Kuwait	Islamic	136	− 0.090	2.225	0.839	98	0.055	1.001	0.179	38	− 0.467	3.903	0.999	− 1.231	(0.220)
	Conventional	136	− 0.123	2.162	(0.402)	98	0.048	0.917	(0.857)	38	− 0.568	3.817	(0.323)	− 1.499	(0.136)
Malaysia	Islamic	167	− 0.038	1.142	1.285	99	− 0.011	0.564	0.451	68	− 0.078	1.662	1.249	− 0.372	(0.710)
	Conventional	167	− 0.074	1.165	(0.200)	99	− 0.023	0.510	(0.652)	68	− 0.148	1.724	(0.215)	− 0.682	(0.495)
Nigeria	Islamic	167	− 0.030	1.286	0.287	124	0.068	1.100	0.013	43	− 0.315	1.698	0.402	− 1.696*	(0.091)
	Conventional	167	− 0.050	1.251	(0.774)	124	0.067	0.917	(0.989)	43	− 0.390	1.888	(0.689)	− 2.086**	(0.038)
Pakistan	Islamic	169	0.131	2.145	0.805	124	0.221	1.383	0.202	45	− 0.116	3.483	0.869	− 0.904	(0.367)
	Conventional	169	0.099	1.812	(0.421)	124	0.214	1.196	(0.839)	45	− 0.217	2.898	(0.389)	− 1.373	(0.171)
Qatar	Islamic	170	− 0.071	1.227	0.381	126	− 0.084	0.615	− 1.194	44	− 0.036	2.195	1.374	0.219	(0.826)
	Conventional	170	− 0.082	1.307	(0.703)	126	− 0.057	0.639	(0.234)	44	− 0.152	2.350	(0.176)	− 0.412	(0.680)
Taiwan	Islamic	159	− 0.010	1.461	− 1.748*	96	0.108	0.609	− 1.463	63	− 0.191	2.194	− 0.976	− 1.268	(0.206)
	Conventional	159	0.048	1.500	(0.082)	96	0.170	0.736	(0.146)	63	− 0.136	2.202	(0.332)	− 1.261	(0.209)
Thailand	Islamic	166	− 0.102	2.166	0.225	96	− 0.021	0.732	0.748	70	− 0.214	3.234	0.060	− 0.563	(0.573)
	Conventional	166	− 0.124	1.990	(0.821)	96	− 0.049	0.664	(0.455)	70	− 0.227	2.974	(0.952)	− 0.568	(0570)
Turkey	Islamic	171	0.234	1.827	2.837***	136	0.209	1.352	2.755***	35	0.330	3.070	1.237	0.347	(0.728)
	Conventional	171	0.041	1.738	(0.005)	136	0.041	1.435	(0.006)	35	0.038	2.631	(0.224)	− 0.009	(0.992)
UAE	Islamic	134	− 0.044	1.501	0.178	79	0.085	0.738	− 0.159	55	− 0.229	2.168	0.236	− 1.195	(0.234)
	Conventional	134	− 0.080	1.928	(0.858)	79	0.101	0.855	(0.873)	55	− 0.342	2.825	(0.813)	− 1.315	(0.190)
Total	Islamic	2416	− 0.006	1.788	1.982**	1641	0.051	0.962	0.793	775	− 0.129	2.828	1.820*	− 2.324**	(0.020)
	Conventional	2416	− 0.042	1.744	(0.047)	1641	0.042	0.894	(0.427)	775	− 0.221	2.783	(0.069)	− 3.484***	(0.000)

***,**,* Represent statistical significance at 1%, 5%, and 10% levels, respectively; Obs, UAE, Is, and Co denote observations, United Arab Emirates, Islamic index, and conventional index, respectively; *p*-values are given in parenthesis

For the period before the pandemic, Panel A_1_ of Table 3 shows that there are significant differences in the average returns between Islamic and conventional indices for Japan and Turkey at 10% and 1% significance level, respectively, but not for the rest of the indices from the other countries. As Panel A_1_ presents, the mean values of the Islamic and conventional stock market returns in Japan are + 0.166 and + 0.141, respectively, which reflects that, on average, the return of Islamic index is statistically higher than the conventional one. Similarly, the mean values of both indices in Turkey are + 0.209 and + 0.041, respectively, meaning that, on average, the return of Islamic index is very strong and statistically higher than the conventional one. Regarding all the countries, however, there is no significant difference between the two means at any significance level for the period before the pandemic.

In terms of the period during the pandemic, Panel A_2_ of Table 3 reports that there are significant differences in the average returns between Islamic and conventional indices only for all the countries together at 10% significance level, but not for the indices from each country. As shown in Panel A_2_, the mean values of the Islamic and conventional stock market returns are − 0.129 and − 0.221, respectively, which reflects that, on average, the returns for both the indices are negative, but the Islamic indices are statistically less negative compared to the conventional ones.

Table 3 also shows the results of sample *t*-tests that explain whether the means of each type of the indices (Islamic and conventional) during the pandemic are statistically different from its means before the pandemic. As presented in the last two columns of Table 3, the mean returns of the Islamic indices during and before the pandemic in Japan are − 0.232 and + 0.166, respectively, which reflects that, on average, the return for the Islamic index during the pandemic is statistically and significantly less than their return before the pandemic at 10% significance level. In Nigeria, it is also found that the returns for both indices, Islamic and conventional, during the pandemic are statistically and significantly less than their returns before the pandemic at 10% and 5% significance level, respectively.

For the total sample, the findings indicate that the mean returns of the Islamic indices during and before the pandemic are − 0.129 and + 0.051, respectively, which reflects that, on average, the returns for the Islamic indices during the pandemic are statistically and significantly less than their returns before the pandemic (at 5% significance level). In contrast, the mean returns of the conventional indices during and before the pandemic are − 0.221 and + 0.042, respectively, which reflects that, on average, the returns for the conventional indices during the pandemic are very strong and statistically significant less than their returns before the pandemic (at 1% significance level).

In summary, the findings from Table 3 show that Islamic indices significantly outperformed the conventional indices over the overall period and during the COVID-19 pandemic period, even when the reported returns are negative. Furthermore, although Islamic indices also outperformed the conventional indices over the period before the COVID-19 pandemic, this is not statistically significant. Importantly, it is found that the returns of each type of indices, Islamic and conventional, decreased significantly during the pandemic period as compared to their returns for the period before the pandemic. However, the impact of pandemic on the Islamic indices return is lower than that on the conventional ones.

**Fig. 1:**
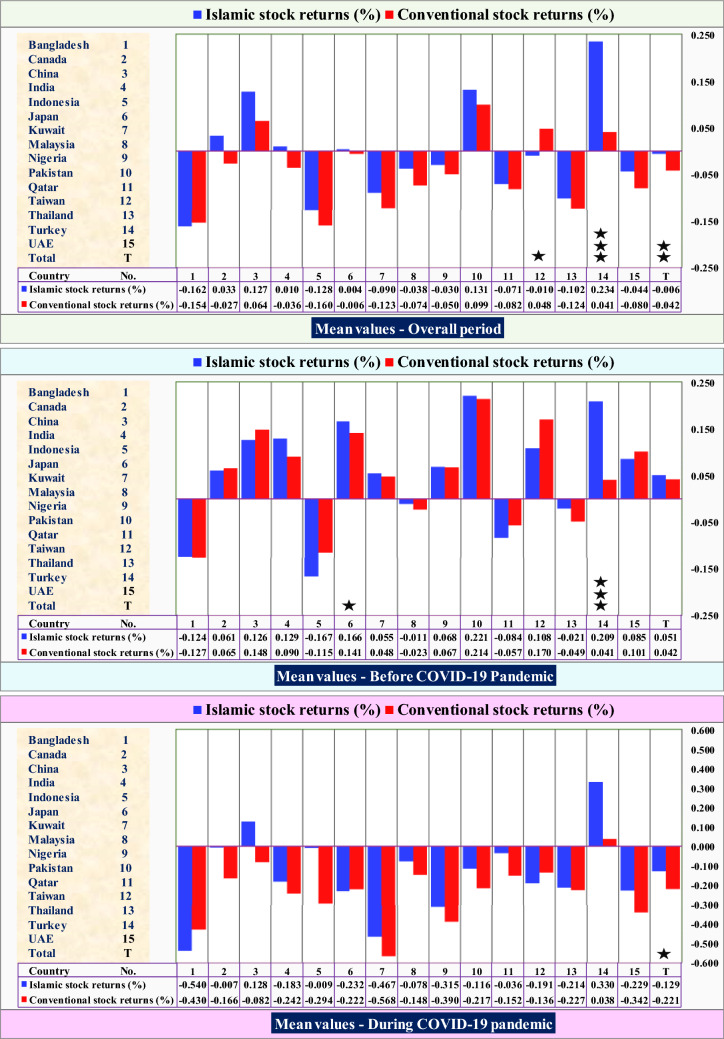
Islamic versus conventional stock market returns (%) for the countries across different periods: overall, before and during COVID-19 pandemic (returns on average). Note: ***, **, * represent *t*-tests statistical significance at 1%, 5%, and 10% levels, respectively

**Fig. 2:**
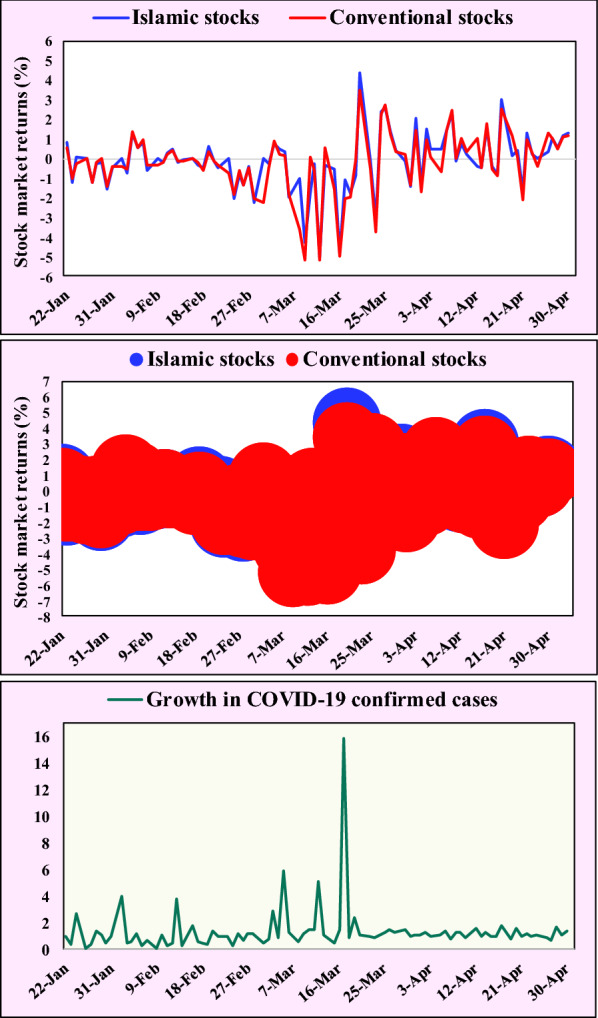
Islamic versus conventional stock market returns (%) for the full sample against the date from the day when 1st COVID-19 case was confirmed and growth in confirmed cases in a country (returns on average)

Figures 1 and 2 also provide a clearer explanation for the differences between the means of return of Islamic and conventional indices for each country over the three main periods, as well as the *t*-test significant levels. In Fig. 1, the Islamic versus conventional stock market indices returns for all the countries across different periods: overall period, before, and during COVID-19 pandemic, are presented. As Fig. 1 presents, the Islamic index from China (Country No. 3), is positive over all the three different periods, while in contrast, the conventional one is positive over the overall period and before the COVID-19 pandemic period and negative during the pandemic. Interestingly, for both indices from Turkey (Country No. 14), they are positive and the Islamic index outperformed the conventional one over all the periods, overall, before, and during the COVID-19 pandemic. This outperformance, however, is strong and significant for the overall period and before the pandemic (at 1% significance level) but not for the period during the pandemic. These findings on Turkey are in line with the empirical findings of Erdoğan et al. [9] who examine the impact of the pandemic on the conventional and Islamic stock markets in Turkey and find that Islamic stock market is more stable to the pandemic shock than the conventional one. Lastly, Fig. 1 presents that Islamic indices outperformed the conventional ones for the overall period and during the pandemic period for all the countries (T) which is statistically significant at 5% and 10% significance levels, respectively.

In a different manner, Fig. 2 also shows the Islamic versus conventional stock market returns for all the countries (full sample) against the date from the day when 1st COVID-19 case was confirmed and the growth in confirmed cases in a country (during COVID-19 pandemic). Figure 2 confirms the above findings, as shown in Table 3 and Fig. 1, that Islamic indices outperformed the conventional ones during the pandemic period for all the countries which is statistically significant at 10% significance level.

Table 4 presents the findings with the panel pooled OLS regression technique for the impact of COVID-19 on Islamic stock market returns (%). The results of estimating Eq. (1), which includes growth in confirmed cases, country-level control variables and weekly fixed-effects dummy variables, show that growth in confirmed cases variable has a negative and weak significant impact on the Islamic stock market returns (at 10% significance level and 0.010 as highest coefficient) for the initial sample and first sub-sample, while its impact is also negative but with modest significance (at 5% significance level and 0.011 as highest coefficient) for the rest of sub-samples, namely second, third, and fourth. However, when replacing country-level control variables with country fixed-effects dummy variables as in Eq. (2), the impact of growth in confirmed cases on Islamic stock market returns remains negative for all the samples but not significant at all.

**Table 4:** Regression results for the impact of COVID-19 on Islamic stock market returns (%)

Panel (A_2_): during COVID-19 pandemic										
Sample	Initial full sample		First sub-sample		Second sub-sample		Third sub-sample		Fourth sub-sample	
	1st case day–April 30		March 12–March 31		March 12–April 10		March 12–April 17 March 12–April 17		March 12–April 30	
Variable	Islamic stock market returns (%)									
	(1)	(2)	(1)	(2)	(1)	(2)	(1)	(2)	(1)	(2)
Growth in confirmed cases	− 0.009*	− 0.010	− 0.010*	− 0.000	− 0.011**	− 0.008	− 0.011**	− 0.008	− 0.011**	− 0.009
	(0.080)	(0.144)	(0.061)	(0.983)	(0.021)	(0.308)	(0.025)	(0.242)	(0.018)	(0.146)
Democratic accountability	− 0.048		− 0.197		− 0.132		− 0.046		− 0.022	
	(0.669)		(0.507)		(0.518)		(0.785)		(0.864)	
Uncertainty avoidance	− 0.001		0.008		0.008		0.003		0.003	
	(0.871)		(0.704)		(0.535)		(0.761)		(0.678)	
Investment freedom	0.009		0.022		0.016		0.013		0.010	
	(0.503)		(0.523)		(0.489)		(0.484)		(0.500)	
Log (GDP)	0.226		0.543		0.517		0.339		0.293	
	(0.386)		(0.426)		(0.253)		(0.364)		(0.304)	
Constant	− 3.478	− 0.284	− 7.192	5.468***	− 9.692	1.230	− 4.457	5.007***	− 3.344	5.382***
	(0.340)	(0.645)	(0.415)	(0.007)	(0.105)	(0.367)	(0.364)	(0.004)	(0.369)	(0.002)
Week fixed-effects	Yes	Yes	Yes	Yes	Yes	Yes	Yes	Yes	Yes	Yes
Country fixed-effects		Yes		Yes		Yes		Yes		Yes
Number of observations	564	564	168	168	263	263	329	329	446	446
Number of countries	15	15	15	15	15	15	15	15	15	15
*R*^2^	0.075	0.089	0.065	0.118	0.077	0.114	0.069	0.098	0.070	0.096

***, **, *Represent statistical significance at 1%, 5%, and 10% levels, respectively; *p*-values are given in parenthesis; Panel (A_2_) represents the period during COVID-19 (the day when 1st COVID-19 case was confirmed in a country – April 30, 2020); VIF values for each model do not indicate multicollinearity problem; 1st case day denotes 1st COVID-19 case was confirmed in a country; March 12, 2020 is the first day when all the 15 countries in the sample reported at least one positive case (see Table 1) as conducted by Topcu and Gulal [21]; The panel pooled OLS regression is employed with the heteroskedasticity robust standard errors

Similarly, Table 5 shows the regression results for the impact of COVID-19 on conventional stock market returns (%). As shown in Table 5, the results of Eq. (1) indicate that growth in confirmed cases variable has a negative and very strong significant impact on the conventional stock market returns (at 1% significance level and 0.016 as the lowest coefficient) for all the samples, the initial sample and the four sub-samples. These results also remain negative and significant for all the samples when estimating Eq. (2) with the exception of the first sub-sample where the impact is negative but not significant. As Eq. (2) of Table 5 shows, growth in confirmed cases variable has a negative and modest significant impact on the conventional stock returns (at 5% significance level and 0.015 as the lowest coefficient) for the initial sample, third, and fourth sub-samples, while its impact is negative with a weak significant (at 10% significance level and 0.015 as coefficient) for the second sub-sample.

**Table 5:** Regression results for the impact of COVID-19 on conventional stock market returns (%)

Panel (A_2_): during COVID-19 pandemic										
Sample	Initial full sample		First sub-sample		Second sub-sample		Third sub-sample		Fourth sub-sample	
	1st case day–April 30		March 12–March 31		March 12–April 10		March 12–April 17		March 12–April 30	
Variable	Conventional stock market returns (%)									
	(1)	(2)	(1)	(2)	(1)	(2)	(1)	(2)	(1)	(2)
Growth in confirmed cases	− 0.016***	− 0.015**	− 0.019***	− 0.009	− 0.019***	− 0.015*	− 0.019***	− 0.015**	− 0.019***	− 0.016**
	(0.008)	(0.021)	(0.007)	(0.361)	(0.005)	(0.065)	(0.004)	(0.035)	(0.004)	(0.015)
Democratic accountability	− 0.010		− 0.041		− 0.036		0.010		0.018	
	(0.921)		(0.891)		(0.859)		(0.950)		(0.881)	
Uncertainty avoidance	0.002		0.011		0.011		0.007		0.006	
	(0.764)		(0.634)		(0.480)		(0.567)		(0.502)	
Investment freedom	0.002		0.018		0.014		0.008		0.005	
	(0.856)		(0.641)		(0.586)		(0.710)		(0.755)	
Log (GDP)	0.070		0.184		0.270		0.135		0.118	
	(0.794)		(0.799)		(0.569)		(0.731)		(0.689)	
Constant	− 1.625	− 0.551	− 3.538	5.082**	− 6.588	1.516	− 2.165	4.645**	− 1.288	5.043***
	(0.665)	(0.264)	(0.701)	(0.017)	(0.282)	(0.284)	(0.671)	(0.014)	(0.737)	(0.006)
Week fixed-effects	Yes	Yes	Yes	Yes	Yes	Yes	Yes	Yes	Yes	Yes
Country fixed-effects		Yes		Yes		Yes		Yes		Yes
Number of observations	564	564	168	168	263	263	329	329	446	446
Number of countries	15	15	15	15	15	15	15	15	15	15
*R*^2^	0.073	0.085	0.044	0.089	0.063	0.099	0.061	0.084	0.064	0.084

***, **, *Represent statistical significance at 1%, 5%, and 10% levels, respectively; *p*-values are given in parenthesis; Panel (A_2_) represents the period during COVID-19 (the day when 1st COVID-19 case was confirmed in a country – April 30, 2020); VIF values for each model do not indicate multicollinearity problem; 1st case day denotes 1st COVID-19 case was confirmed in a country; March 12, 2020 is the first day when all the 15 countries in the sample reported at least one positive case (see Table 1); The panel pooled OLS regression is employed with the heteroskedasticity robust standard errors

The results of Tables 4 and 5 suggest that both stock market indices, Islamic and conventional, are negatively impacted by the growth in COVID-19 confirmed cases. However, the power of the impact is different, while the impact is weak on the Islamic stock markets it is very strong on the conventional ones.

To simplify the discussion, Table 6 summarizes the results of panel pooled OLS regression for the impact of COVID-19 on Islamic and conventional stock market returns for the initial full sample, Panel A_2_, and the four sub-samples. Further, it summarizes the paired *t*-test of Islamic stock market returns versus conventional ones during the COVID-19 pandemic for the initial full sample, besides presenting further analysis for the four sub-samples.

**Table 6:** Summary of regression and paired *t*-test analysis for the Islamic and conventional stock market returns during COVID-19 pandemic

Panel (A_2_): during COVID-19 pandemic							
Analysis technique	Panel pooled OLS regression with the heteroskedasticity robust standard errors for the impact of growth in COVID-19 confirmed cases on stock market returns				Paired *t*-test of stock market returns (Islamic vs. conventional)		
Variable	Islamic stock returns (%)		Conventional stock returns (%)		Islamic stock returns (%)	Conventional stock returns (%)	*t*-value
	(1)	(2)	(1)	(2)	Mean	Mean	(*p*-value)
Initial full sample	− 0.009*	− 0.010	− 0.016***	− 0.015**	− 0.129	− 0.221	1.820*
1st case day–April 30	(0.080)	(0.144)	(0.008)	(0.021)			(0.069)
*Further analysis: Eliminating the initial full sample into four sub-samples based on period*							
First Sub-sample	− 0.010*	− 0.000	− 0.019***	− 0.009	− 0.477	− 0.692	1.627
March 12–March 31	(0.061)	(0.983)	(0.007)	(0.361)			(0.105)
Second sub-sample	− 0.011**	− 0.008	− 0.019***	− 0.015*	− 0.073	− 0.249	1.911*
March 12–April 10	(0.021)	(0.308)	(0.005)	(0.065)			(0.057)
Third sub-sample	− 0.011**	− 0.008	− 0.019***	− 0.015**	0.027	− 0.130	2.053**
March 12–April 17	(0.025)	(0.242)	(0.004)	(0.035)			(0.040)
Fourth sub-sample	− 0.011**	− 0.009	− 0.019***	− 0.016**	0.145	− 0.021	2.773***
March 12–April 30	(0.018)	(0.146)	(0.004)	(0.015)			(0.005)

This table summarizes the results of panel pooled OLS regression for the impact of COVID-19 on Islamic and conventional stock market returns (%) for the initial full sample (Panel A_2_) and the four sub-samples as shown in Tables 4 and 5. Further, it summarizes the paired *t*-test of Islamic stock market returns versus conventional stock market returns during COVID-19 pandemic for the initial full sample (Panel A_2_) as shown in Table 3, besides presenting the further analysis for the four sub-samples; ***, **, * represent statistical significance at 1%, 5%, and 10% levels, respectively; *p*-values are given in parenthesis; Panel (A_2_) represents the period during COVID-19 (the day when 1st COVID-19 case was confirmed in a country—April 30, 2020); VIF values for each model do not indicate multicollinearity problem; 1st case day denotes 1st COVID-19 case was confirmed in a country; March 12, 2020 is the first day when all the 15 countries in the sample reported at least one positive case (see Table 1)

Regarding the additional *t*-tests for the four sub-samples, Table 6 depicts that the Islamic indices significantly outperformed the conventional ones for the second, third, and fourth sub-samples at 10%, 5%, and 1% significance levels, respectively, while the Islamic indices outperformed their counterparts for the first sub-sample, but the difference is not significant. Moreover, and more interestingly, Table 6 shows that the mean values of the Islamic stock market returns are − 0.477, − 0.073, + 0.027, and + 0.145 for the four sub-samples during the pandemic period, respectively, indicating that the returns of the Islamic indices have gradually improved and begun to be positive instead of negative by April 17, i.e. the third sub-sample. In contrast, the results reveal that the mean values of the conventional stock market returns are − 0.692, − 0.249, − 0.130, and − 0.021 for the four sub-samples during the pandemic period, respectively, meaning that although the returns of conventional indices have gradually improved, it remains negative over all the periods.

In general, these findings are in line with that of Topcu and Gulal [21], who found that the negative impact of pandemic on emerging stock markets has gradually fallen and started to taper off by mid-April, 2020. Overall, the findings are almost consistent with Sherif [19] who found that the COVID-19 pandemic negatively but insignificantly impacts the performance of the UK Dow Jones Islamic index, while in contrast, the pandemic strongly and significantly impacts the performance of its UK conventional counterpart.

## Conclusion

This study investigates the impact of COVID-19 on Islamic versus conventional stock markets returns using daily data from 15 countries for the period between September 01, 2019, and April 30, 2020, which covers two main periods (before and during COVID-19) and over four sub-periods during COVID-19. To analyse the data, the study employs sample *t*-tests and panel pooled OLS regression. The *t*-tests findings reveal that Islamic indices significantly outperformed the conventional ones over most of the periods. Importantly, findings also suggest that returns of both indices, Islamic and conventional, have gradually improved. However, the returns of Islamic indices begin to be positive instead of negative by mid-April, 2020, while returns of conventional ones remain negative over all periods. Further, the regression findings show that both stock indices respond negatively to the COVID-19; however, this negative impact is weak on the Islamic markets and very strong on the conventional ones. Based on the findings, it can be inferred that Islamic stock markets would offer a better hedge against COVID-19 crisis than the conventional ones.

The results of the study have important implications for investors, policy makers as well as researchers. For stock markets investors who seek for a more resilient return on investment, this study provides evidence that the performance of Islamic indices is generally better than the conventional ones, especially during the COVID-19 pandemic crisis. Further, investors may consider investing in Islamic and/or conventional indices components stocks from Turkey during crisis or non-crisis periods, and the investment in Islamic indices stock components are the most recommended. For policy makers, this study may assist them for policy implementation during the pandemics. In general, the study provides an empirical evidence for policy makers and researchers to understand the behaviour of Islamic and conventional stock markets during COVID-19 crisis.

Despite its extensiveness, the sample of this study is only 15 pairs of Islamic and conventional indices; hence, it is more insightful for future studies to investigate a wider range of indices made available in the markets across countries. Moreover, future research should also focus on the Turkish stock market and investigate the impact of COVID-19 on wider pairs of Islamic and conventional indices, besides comparing their performance over crisis and non-crisis periods. This can provide a much clearer evaluation for the Turkish stock markets based on a large sample instead of only two pairs of indices like in this study.

## Data Availability

It will be provided on request.

## Appendix 1

See Table 7.

**Table 7:** Variable definitions and the data source

Variable	Definition	Reference	Data source
*Dependent variables*			
Islamic stock market returns (%)	The daily change in the Islamic stock index of a country which is calculated as: (Index value_t_ − Index value_t − 1_/Index value_t − 1_)** × **100	Ashraf [4, 5]	www.investing.com
Conventional stock market returns (%)	The daily change in the conventional stock index of a country which is calculated as: (Index value_t_ − Index value_t − 1_/Index value_t − 1_) × 100		
*Main independent variable*			
Growth in confirmed cases	The daily growth rate of COVID-19 confirmed cases for a country which is calculated as ((Cases_t_ – Cases_t − 1_)/Cases_t − 1_)	Ashraf [4, 5]	Authors calculation with data from the website of EU Open data portal
*Control variables*			
Democratic accountability	Democratic accountability index represents the quality of political institutions. The higher values show higher democratic accountability and vice versa	Ashraf [4, 5]	International country risk guide database
Uncertainty avoidance	Uncertainty avoidance index is used to control for cross-country differences in the level of uncertainty aversion in investors. Index values range from 0 to 100 where higher values represent higher national-level uncertainty avoidance and vice versa	Ashraf [4, 5]	Hofstede et al. [13]
Investment freedom	Investment freedom index measures the level of freedom to invest in financial markets. It is used to control for stock market liberalization. The index ranges from 0 to 100 where higher values represent higher investment freedom and vice versa	Ashraf [4, 5]	Heritage foundation [11]
Log (GDP)	The natural logarithm of annual gross domestic product (GDP) of each country. It measures the level of economic development	Ashraf [4, 5]	The world economic outlook database, international monetary fund website [20]

## Abbreviations

OLS: Ordinary least squares
UK: United Kingdom
GDP: Gross domestic product
WHO: World Health Organization
Obs: Observations
UAE: United Arab Emirates
Is: Islamic index
Co: Conventional index

## Footnotes

1 DJIM: The Dow Jones Islamic Market Asia/Pacific index; DJCA: The Standard and Poor’s (S&P) Dow Jones Composite Average stock index.

2 These indices are the Dow Jones world stock market index, the Dow Jones Islamic stock market index, the ICE BofA world Bond Market Index and the Dow Jones World Sukuk Index.

3 These countries were included as they have available data on both Islamic and conventional stock market indexed on the website.

4 This study selects a relatively short period pre and post the pandemic to avoid the occurrences of any exogenous factors that may influence the stock markets and then bias the results, following Okorie and Lin [15].

5 This platform is one of the top three global financial websites [18].

6 This study uses the number of COVID-19 confirmed cases as an indicator for the COVID-19 and neglects the number of COVID-19 deaths. According to Erdem [8], the number of COVID-19 confirmed cases data is an early warning signal and offers an idea of the coming death rates. Thus, there is no need to get the number of deaths.
